# Built environment (BE) and cancer: a systematic review of the BE’s impact during the treatment journey and patient outcomes

**DOI:** 10.1007/s10552-026-02132-5

**Published:** 2026-05-26

**Authors:** Rafael Salas Carretero, Matteo Perillo, Antonello Lorenzini, Paolo Boffetta

**Affiliations:** 1 Department of Medical and Surgical Sciences, University of Bologna, Bologna, Italy; 2 Department of Biomedical and Neuromotor Sciences, University of Bologna, Bologna, Italy; 3 National Institute of Biosystems and Biostructures INBB, Rome, Italy; 4 Stony Brook Cancer Center, Stony Brook University, Stony Brook, NY USA; 5 Maria Sklodowska-Curie National Research Institute of Oncology (MSCNRIO), Warsaw, Poland

**Keywords:** Built environment, Urban environment, Cancer, Cancer-outcome, Cancer infrastructure, Systematic review

## Abstract

The role of the built environment (BE) in cancer research has been extensively explored in prevention, focusing on its influence as a determinant of exposure to carcinogens and a lifestyle shaper, particularly through geographic accessibility to resources like transportation, healthy food, and diagnostic centers. However, the BE’s role and influence during the treatment period and its connection with patient outcomes remain comparatively understudied. This systematic review employed the CDC definition of BE, following PRISMA guidelines and AI-driven software (ASReview) for a thorough and efficient literature review. A total of 31 articles were identified (27 database, 4 snowballing) that examined the BE’s role during the treatment phase. These studies investigated diverse BE characteristics including urban features (e.g., transportation, green environment, food environment) and architectural features (e.g., design, aesthetics) during the cancer patient treatment journey. Among the included studies, the most analyzed outcomes were Physical Activity (PA) (11 articles), followed by survival and mortality (9 articles). Findings revealed positive associations between specific BE characteristics—such as green spaces, accessibility, transportation options, and aesthetics—and cancer patients’ physical activity levels, quality of life, and mental health status; however, findings are inconsistent. These results underscore the potential of integrating urban and architectural BE analysis to enhance the psychosocial dimensions of cancer treatment, improve healthcare delivery, and open new pathways for research strategies.

**Supplementary Information:**

The online version contains supplementary material available at 10.1007/s10552-026-02132-5.

## Background

Cancer is one of the most extensively researched diseases, receiving one of the highest levels of funding [1, 2] due to its significant burden on public health, with high rates of incidence and mortality [3]. This investment has driven the global expansion of OIs in recent years [4]. These integral components of the built environment (BE), not only provide the setting for continuing the advance of research in cancer biology and treatment [5] but also aim to improve the spaces where patients receive care as these infrastructures are part of a broader BE framework that influences patient experiences, access to care, and outcomes.

Parallel to this, interest in the influence of the BE on general’s population health has grown [5–7], and it has been also seen reflected on the increase on research about the association between cancer and BE. Aiming to address better prevention strategies, this research has largely centered on viewing the BE as both a place of exposure to carcinogens and a lifestyle shaper through economic, geographic, and demographic factors [8]. Studies have highlighted BE’s role in toxin exposure and lifestyle-related cancer risks, establishing its influence on cancer onset [9, 10].

However, the role of BE during the cancer treatment journey, when OI is most directly experienced, has been comparatively overlooked [11]. For patients, this journey that can span from 6 to 18 months for curable cases to a lifetime for incurable conditions [12], this period is marked by frequent access to OIs while dealing with physical, psychological, social, and economic challenges and needs [13]. At this point, these facilities become pivotal during treatment, raising the questions: What is the role that the BE plays during this journey? *How does the BE shape the patient experience and outcomes during this journey?*

The primary goal of this review was to identify BE variables relevant during the extended treatment period, a phase where oncology infrastructure (OI), the patient’s residence, and the environments surrounding is most directly encountered by patient. Findings were intentionally grouped into three categories of BE variables:

1. Density-related variables (DRV): reflecting intrinsic socio demographic characteristics such as population density and housing crowding.
2. Urban-related variables (URV): capturing external features influencing access and mobility, including transportation systems, walkability, food and retail environments, and proximity to health and recreational facilities.
3. Architecture-related variables (ARV): encompassing design, aesthetics, green spaces, and indoor environmental qualities within treatment or residential settings.

This categorization enabled a more patient-centered analysis of the BE by linking general environmental characteristics to those most relevant within and around OIs. Recent advances in Geographic Information Systems (GIS) and artificial intelligence (AI) [14] offer significant potential for detailed mapping and analysis of the environments that patients navigate and advance research on its impact. These technologies help bridge the gap between prevention-focused BE research and the understanding of BE’s role in supporting patients throughout treatment.

## Methods

We carried out a systematic review of the scientific literature investigating the role of BE during the treatment journey and its connection with patient outcomes. Each phase of the study was conducted, and its partial results reported according to the “Preferred Reporting Items for Systematic Reviews and Meta-Analysis” (PRISMA) checklist [15]. Due to the heterogeneity of outcomes, BE variables and measures of association, no meta-analysis or quantitative synthesis was performed.

### Search strategy

A systematic search of relevant papers was carried out in the following databases: PubMed, Science Direct, Journal of Urban Planning and Development, Scopus, SAGE publications, and ASCE—American Society of Civil Engineers.

The research was carried between May and June 2023, and the search query was built to find articles published between January 2008 and April 2023 which comprised in their title, abstract, or keywords the words “cancer” and “built environment,” or “transport,” or “urban,” or “neighborhood” or “public transport,” or “urbanism” in combination with “tumor-free” or “tumour free” or “outcome” or “results.” Reference lists of the retrieved articles were inspected manually to identify any relevant papers through snowballing.

No language restrictions were applied, all identified records were in English and integrated and deduplicated using EndNote Web. The time limit (2008–2023) was selected to capture recent research following major developments in OI design and identify the use of spatial and AI-based analytical tools for environmental health research.

Importantly, the search strategy was intentionally broad to encompass studies that may not use the term “oncology infrastructure” explicitly but that analyzed BE components directly relevant to the environments patients inhabit or navigate during the treatment phase such as example, hospitals, residential neighborhoods, and community facilities associated with cancer care access and recovery.

### Inclusion criteria

This systematic review examines the role of BE during the cancer treatment journey, focusing on how BE characteristics influence patient outcomes across different cancer types. The review targets original studies investigating BE-outcome relationships in cancer patients (CPs) or survivors, emphasizing tertiary prevention outcomes such as cancer mortality, relapse, and physical or psychological well-being.

To align with this treatment-phase focus, eligibility criteria were framed to include studies whose results take into consideration oncology-related environments’ characteristics, whether at the architecture scale (e.g., home, hospitals, clinics, recovery centers), or urban scale (e.g., local support environments, accessibility and mobility networks affecting care access).

Studies were included only if they identified at least two variables, analyzed them at one of these scales, and explicitly related them to CP outcomes, including pre-defined archetypes encompassing multiple BE features. Studies focusing solely on single environmental features (e.g., green areas alone) or purely geographic typologies (e.g., urban–rural) were excluded to ensure analysis captured the complex interplay of multiple environmental factors.

The exposure of interest is the BE, defined according to the Centers for Disease Control and Prevention as “The physical makeup of our living, learning, working, and recreational spaces—homes, schools, businesses, streets, sidewalks, open spaces, and transportation options” [16]. Non-original works, reviews, commentaries, and abstracts were not included. No geographic limitations were applied. Table 1 outlines the inclusion criteria.

**Table 1:** Inclusion criteria

Inclusion criteria	
Publication year:	2008 – 2023
Country of publication:	No restrictions
Topic of the article:	BE and health/treatment outcomes
Population:	Cancer patients/survivors
Population size:	No restrictions
Population age:	18 years old and older
Ethnicity:	No restrictions
Type of cancer:	No restrictions
Cancer stage:	No restrictions
BE definition:	Physical and man-made makeup of the environment
Type of BE variables:	Architectural or Urban
Minimum # of variables:	Two (2)

### Studies selection

After deduplication, we conducted a two-phase screening: first reviewing titles and then analyzing abstracts. Screening was performed using ASReview (v1.0rc0) [17], an open-source machine learning tool to screen and label large dataset. ASReview employs natural language processing and active learning to iteratively suggest relevant articles based on reviewers’ decisions, thus prioritizing important papers and saving time. The full-text screening involved assessing all articles deemed potentially relevant from the title and abstract review to make the final inclusion decisions for the review.

In both phases, two authors (R.S.C. and M.P.) worked together to label each record as relevant or irrelevant based on the inclusion criteria. Any disagreements were resolved through discussion, including the other authors (A.L., P.B.).

### Data extraction

A pre-defined, customized, and original spreadsheet was used to extract and collect data from the selected papers. Two authors (R.S.C. and M.P.) performed the extraction, capturing both qualitative and quantitative information. Qualitative data included the first author’s name, year of publication, country, study design, interdisciplinary collaboration, BE definition type, cancer type, BE features, prevention level, outcome domains, and, when available, sociodemographic characteristics (e.g., gender, ethnicity, NSES). Quantitative data included scope, sample size, and significant results quantifying the association between BE and cancer outcomes.

Articles were grouped by the scale of their area of analysis, as this affected the BE variables they used. Smaller areas focused on specific variables, while larger areas considered more general ones. Each article was classified as follows:

- Metropolitan area: analyzed city-wide characteristics, irrespective of patient residence.
- Residence area: focused on the characteristics of the patient’s neighborhood.
- Infrastructure: examined oncology infrastructures (OIs) or home.

### Risk of bias assessment

The risk of bias related to sample selection, comparability, and exposure ascertainment for all included articles was jointly assessed by two authors (R.S.C., M.P.) using the Newcastle–Ottawa Scale (NOS) for observational studies. Although the tool is designed for case–control and cohort studies, the cohort version was also used to evaluate cross-sectional studies and randomized controlled trials. In these cases, questions 4, 7, and 8 were omitted. Questions 4 and 7 were also skipped for cohort studies with measurable outcomes (e.g., BMI, PA, QoL) rather than events (e.g., death, cardiovascular events). The assessment was performed per analysis, so some papers were assessed twice for different analyses. Case and ecological studies were excluded from the assessment.

## Results

### Studies selection

**Fig. 1:**
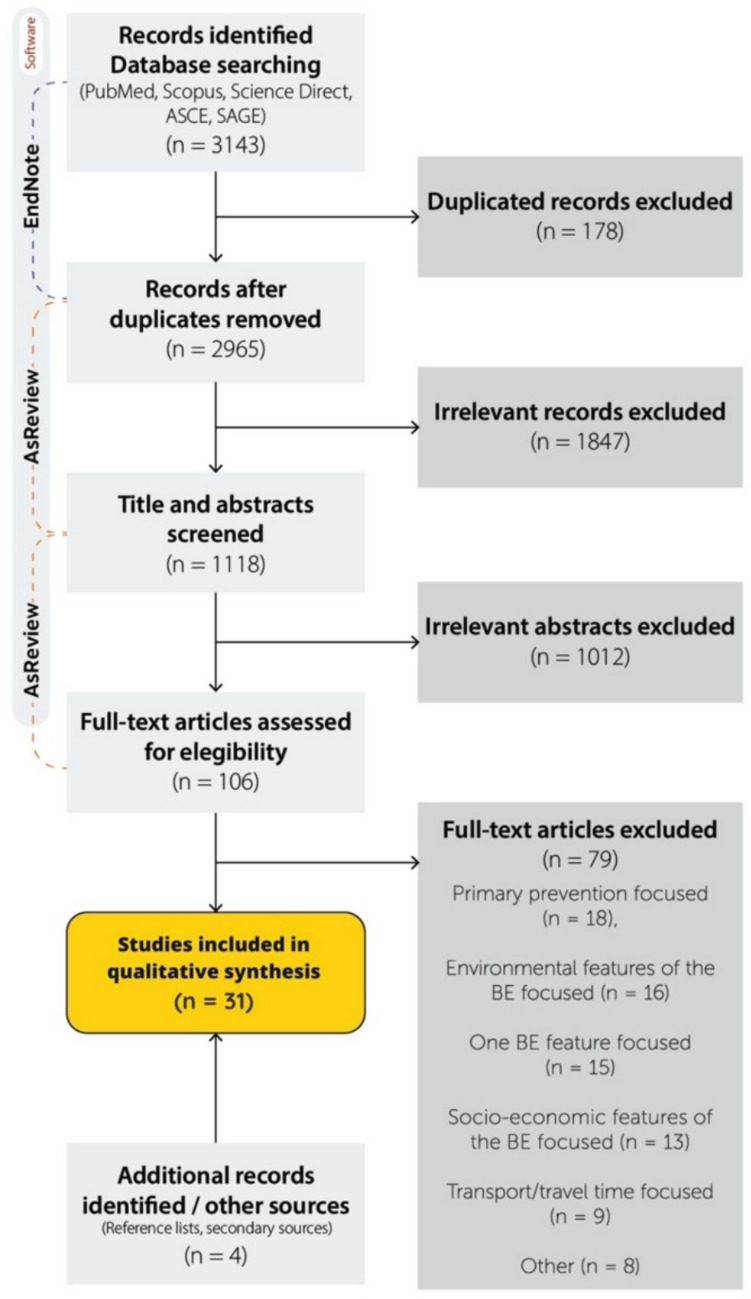
Systematic review process elaboration: authors

The search in the repositories yielded 3143 articles. After duplicate removal (*n* = 178), 2965 articles were loaded into AS Review for the screening. A total of 1847 articles were considered irrelevant based on a title analysis. The remaining 1118 articles underwent abstract screening, during which 106 articles were considered potentially eligible for review. Of these, 79 were excluded after full-text screening. Ultimately, 27 articles met all the inclusion criteria, and an additional 4 articles were identified through snowballing, all of which were included in the review. The PRISMA flowchart in Fig. 1 summarizes the selection and exclusion process.

### Characteristics of included articles

The relevance of the topic is highlighted by the fact that 17 articles (55%) were published within the last four years (2020–2023). In terms of interdisciplinarity, 18 articles (58%) were authored exclusively by individuals from medical fields, while 10 (32%) featured collaborations with non-medical fields. Among these, three articles involved contributors from architecture and urbanism. There were also contributions from kinesiology, sports science, and other diverse disciplines (Table 2).

**Table 2:** Summary of characteristics of included studies

Studies characteristics			
Category	*n*	%	Article reference
Publication year			
2008–2009	1	3	8
2010–2014	2	6	14, 16
2015–2019	11	35	1, 5, 6, 12, 13, 17, 23, 24, 26, 30, 31
2020–2023	17	55	2–4, 7, 9–11, 15, 18–22, 25, 27–29
Study population			
Less than 50	5	16	6, 8, 12, 18, 28
50–1000	10	32	2, 9, 10, 13, 16, 20, 22, 27, 30, 31
1000–10,000	8	26	1, 3, 5, 7, 14, 21, 23, 24
10,000–100,000	2	6	19, 26
100,000 and more	2	6	4, 25
Non-specified	1	3	29
Publication country			
USA	22	71	1–7, 9, 10, 13, 14, 19–28, 31
Canada	5	16	8, 15, 17, 18, 30
Australia	1	3	16
The Netherlands	1	3	11
Belgium	1	3	12
China	1	3	29
Study design			
Quantitative	27	87	1–5, 7, 9–11, 13–27, 29–31
Qualitative	4	13	6, 8, 12, 28
Interdisciplinarity			
Not interdisciplinary/only medical-r.f.*	18	58	1–8, 14, 16, 19–26
Kinesiology-r.f.*	4	13	15, 17, 27, 30
Sports, exercise and physical educ.-r.f.*	4	13	11, 17, 18, 30
Architecture & urbanism-r.f.*	3	10	10, 11, 29
Geography & environmental-r.f.*	2	6	28, 30
Sociology-r.f.*	1	3	13
Engineering, Mathematics and informatics-r.f.*	1	3	31
Only architecture and urbanism-r.f.*	2	6	9, 12
Cancer type			
Breast cancer only	13	42	1, 3, 8, 13, 14, 19, 20, 22–26, 31
Prostate cancer only	4	13	4, 5, 17, 18,
Colon cancer only	1	3	16
Skin cancer only	1	3	28
Lung cancer only	1		29
Kidney cancer only	1	3	30
Multiple	10	33	2, 6, 7, 9, 10–12, 15, 21, 27
Ethnicities definition included			
African American/Non-Hispanic (NH)/Black	14	45	1, 2, 4, 5, 14, 19–26, 31
Asian American/Pacific Islander (API)/NH Api	8	26	1, 2, 14, 19, 23–26
White/NH White	13	42	1, 2, 4, 5, 9, 10, 14, 19, 23–26, 28
Hispanic	12	39	1, 2, 4, 5, 7, 14, 19, 23–26, 31
Arab	1	3	25
Defined as non-White/others	5	16	2, 9, 10, 24, 28
Non-specified	11	35	6, 8, 11, 12, 15–18, 27, 29, 30
Data collection scope			
Residence neighborhood	1	3	30
Residence city	11	35	2, 5, 6, 7, 17, 21–23, 28, 29, 31
Residence state	12	39	1, 3, 4, 8, 14, 16, 19, 20, 24–27
Multi-state	1	3	18
National	5	16	9, 10, 11, 13, 15
International	1	3	12
Area of analysis			
Metropolitan area	1	3	8
Residential area	30	97	1–11, 13–31
Infrastructure	4	14	7, 8, 12, 27

r.f.* related fields, *NH* non-Hispanic, *API* Pacific islander

The study populations varied significantly. One article (4%) focused on patients living in the same neighborhood, 11 (35%) on those residing in the same city, and 12 (39%) on patients from different cities within a state. Only one article (3%) considered patients at an interstate level, while five (16%) had national samples, and one (3%) examined an international sample. Geographically, most articles (87%) focused on the area of residence, and only one article focused exclusively on the BE variables related to infrastructure.

The study designs were diverse, with seven retrospective cohort studies (23%), five prospective cohorts (16%), and ten cross-sectional studies (32%). The remaining articles included case–control studies, multiple study designs, one randomized controlled trial, and one ecological study. Additionally, 15 articles reused data from previous studies, demonstrating the potential for data reuse in this field.

Regarding the types of cancer analyzed, 21 articles focused on a specific type of cancer, with breast cancer being the most common (13 articles), followed by prostate cancer (4 articles). Other types, including colon, kidney, skin, and lung cancer, were each studied in one article. Meanwhile, 10 articles did not specify the type of cancer.

The main Urban variables identified across the studies included transport and traffic (28 articles), green areas/nature (20 articles), street connectivity (18 articles), recreational environment (16 articles), and food environment (14 articles). Architectural variables were also explored, with 18 articles addressing aesthetics variables, 8 the house environment, and 3 each on physical accessibility/safety and design-related factors. Density variables were identified in 20 articles.

**Fig. 2:**
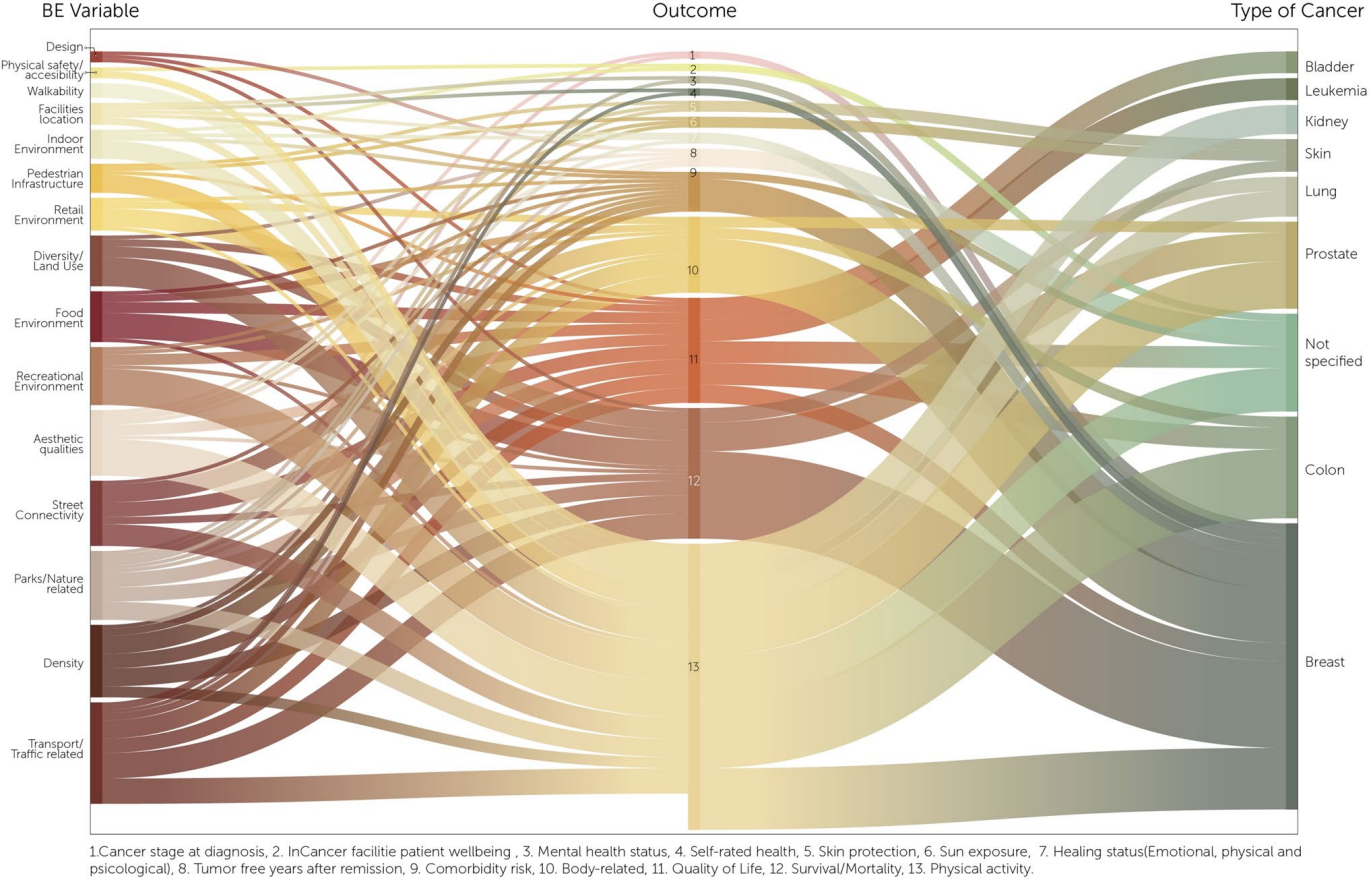
Relation between variables/outcome/type of cancer

Finally, Physical activity was the most analyzed outcome (11 articles), followed by survival and mortality (9 articles). Other outcomes identify were weight-related outcomes, QoL, comorbidities, skin exposure and protection, cancer stage at diagnosis, healing status, tumor-free years after remission, and well-being in cancer facilities. Figure 2 provides a visual summary of the associations between BE variables, health outcomes, and cancer types.

### Observed associations between BE variables and cancer outcomes

The review of observed associations between the BE and cancer outcomes identified Physical Activity (PA) and Survival/Mortality as the most studied outcomes. A significant and consistent theme across the results points to the detrimental effects of high-density [18–20] and urban disorder characteristics on patient health. Specifically, high population density and related factors like household crowding [18] were frequently associated with negative health outcomes, including poorer survival after breast cancer diagnosis [19, 20] and lower physical composite scores (PCS) [21] for Quality of Life (QoL) [14, 21, 22].

Regarding survival, the analysis of Neighborhood Archetypes showed that “inner city” and “Hispanic small town” classifications correlated with a higher mortality risk for prostate CP [21], suggesting that cumulative socioeconomic and urban disadvantages affect prognosis. Furthermore, areas with high traffic density, an unfavorable retail food environment index (RFEI) [23, 24], and increased household crowding [18, 20, 25] were consistently linked to a higher risk of death.

Conversely, specific urban resources and architectural variables demonstrated positive associations, often acting as behavior modifiers to support psychosocial well-being and PA [26–28]. The review emphasized that aesthetic features of the BE, such as the presence of trees, shade, sidewalk quality, and landscape maintenance, significantly influenced patients’ willingness to engage in outdoor PA participation [22, 26, 29] and contributed to better skin protection behaviors for skin cancer survivors [30].

Furthermore, the proximity and quality of blue–green spaces and vegetation cover were negatively correlated with lung cancer mortality [31], while high-quality sidewalks [27, 30, 32] and a lack of abandoned buildings [32] were linked to improved QoL and emotional well-being. Crucially, the internal characteristics of the OI itself were found to be impactful: physical accessibility, safety, and the indoor environment of cancer centers significantly influenced patient emotions and affected psycho-spiritual well-being during the treatment journey [33]. Lastly, specific elements of neighborhood context (including social stressors, crime, and public services) were significant predictors of self-rated QoL and health [14] and were linked to increased psychological difficulties and poorer mental health status when neighborhood stress levels were high [34, 35].

Table 3 summarizes the associations between the BE variables (from Sect. "Data extraction") and the 14 analyzed outcomes, ordered by the number of correlations with BE variables.

**Table 3:** Results of the association between BE variables and cancer outcomes

Analyzed outcome	No of articles	No of variables	Associated BE variables	Main findings/associations
Cancer stage at diagnosis	1	2	Neighborhood aesthetics, green areas/nature environment	Patients in areas with less physical disorder and more green areas had lower odds of late-stage diagnosis and longer breast cancer-specific survival [36]
In-facility patient well-being	1		Indoor environment, physical accessibility/safety	Physical features of cancer centers significantly influenced patient emotions, imposed physical limitations, and impacted psycho-spiritual well-being. [33]
Skin protection and sun exposure	1	3	Pedestrian infrastructure, aesthetics, green areas	Trees, shade, sidewalks, materials, and maintenance were key factors for enhancing skin protection and reducing sun exposure for skin cancer survivors [30]
Healing status	1		Indoor environment, facilities location, green areas	A well-designed home and community spaces, including nature and green areas, were crucial for recovery and overall healing [34]
Tumor-free years	1	5	Residential BE, travel distance	Results show that travel distance to cancer facilities does not reliably predict treatment receipt. However, patients traveling farther to radiotherapy centers tend to have more tumor-free years, supporting findings that longer travel may enhance access to high-quality care despite challenges [37]
Mental health status	1	6	Cluster: Neighborhood context (housing, environment, transportation, public services, crime, violence)	Higher neighborhood stress levels were linked to increased psychological difficulties and poorer mental health, with variations across ethnic groups [21]
Self-rated health	1		Cluster: Neighborhood context (same as above)	Neighborhood context variables were significant predictors of self-rated health [35]
Body-related outcomes (BMI, Obesity)	3	7	Density, Restaurant environment, Fast-food restaurant ratio, Neighborhood Walkability index (NWI), access to public transit, bus-stop density, street connectivity	Among Latinas, higher neighborhood crowding was strongly associated with obesity. For non-Latina white women, lower nSES was consistently linked to higher obesity prevalence. Overall, neighborhood attributes were related to both obesity, but the direction and magnitude of associations varied substantially across racial and ethnic groups [38] Low nSES, high minority composition, high traffic density, high prevalence of commuting by car, and a higher number of fast food restaurants were independently associated with higher odds of overweight or obesity [39]. A higher Neighborhood Walkability Index (NWI) was associated with lower BMI in cancer survivors living in metropolitan areas. [40]
Risk of comorbidities	3	9	Traffic density, psychosocial stressors, neighborhood crime, racial/ethnic composition	Neighborhoods with chronic psychosocial and environmental stressors increase cardiovascular disease (CVD) risk, particularly for women near high-traffic areas. Factors like neighborhood crime and racial/ethnic composition also correlate with elevated CVD risk [23] Higher odds of SARS-CoV-2 infection are linked to neighborhoods with overcrowded households, larger average household sizes, and increased population density [18] Neighborhood context significantly predicts self-rated health and mental health among cancer survivors [35]
Quality of Life (QoL)	4	10	Population density, Street connectivity, Recreational facilities, Unhealthy restaurants ratio, Traffic density, crowding, Rental properties %, non-single-family units%, Perceived accessibility, and Distance to transit	High population density, street connectivity, recreational facilities, unhealthy restaurants, traffic density, crowding, rental properties, and non-single-family units were associated with lower physical composite scores (PCS) and QoL. Only rural residence was linked to lower PCS in an adjusted model [21] Self-reported QoL was mainly influenced by perceived accessibility, distance to transit, and population density, with negative emotions regarding density affecting QoL [14] Abandoned buildings and high-quality sidewalks were linked to better QoL and emotional well-being. Sidewalk quality was the only factor associated with QoL in an adjusted model [32] Perceived stress related to neighborhood disorder decreased significantly after adjustments, with SES and health behaviors influencing perceptions of the BE and stress levels [22]
Survival/Mortality	9	11	Neighborhood archetypes (NArch), Retail food environment index (RFEI), Neighborhood recreation environment index (REI), Household crowding, Population density, multi-family housing density, Business count, Traffic density, Alcohol and tobacco facilities density, Catering facilities density, and Blue-green space and vegetation cover	NArch: No single neighborhood attribute influenced cancer survival; however, “inner city” and “Hispanic small town” NArchs correlated with higher mortality risk for prostate CPs [31] RFEI: Higher mortality risk was linked to unfavorable RFEI and REI, with increased household crowding also contributing to higher death risk for prostate CPs [24] Densely populated neighborhoods were associated with poorer survival after breast cancer diagnosis Latina women in high multi-family housing neighborhoods experienced higher mortality rates, while more businesses in the area were linked to lower breast cancer mortality [38] Increased physical disorder correlated with shorter survival among women with early-stage breast cancer, depending on tumor factors [36] Factors like total business count, housing crowding, urban/rural categorization, population density, public transportation, restaurant environment, traffic density, and hospital socioeconomic composition were linked to obesity and breast cancer mortality [25] A significant association exists between residential density and lung cancer mortality, with lower associations for alcohol and tobacco facilities, catering facilities, bus stops, and road intersections. In contrast, blue-green space, distance to the river, and vegetation cover are negatively correlated with lung cancer mortality [31]
Physical Activity (PA)	11	13	Aesthetics, Sidewalk, Retail shops, home equipment, Rental %, Traffic density, Restaurants, Bike infrastructure, Recreation facilities, Density, Diversity, Parks	Skin Cancer: Aesthetic BE features such as shade, trees, ground cover, sidewalk quality, and landscape maintenance, significantly influence outdoor PA participation [30] Kidney Cancer: No objective BE features predicted meeting PA guidelines; however, perceived proximity to retail shops was significant [41]. Aesthetics, land diversity, and physical safety were important for motivating PA [29] Breast Cancer: Lack of facilities and space increased the odds of not meeting PA guidelines, while higher renter rates and inadequate facilities contributed to lower PA levels [42] Women in neighborhoods without parks had better survival rates if NSES was considered. Lower traffic density and a higher variety of healthy restaurants were independently associated with meeting PA recommendations [19] Colorectal CPs: Barriers to PA included a lack of suitable facilities and unsafe environments, with physical BE features being the least significant [43] Prostate Cancer: Neighborhood aesthetics and sidewalks were the strongest predictors of moderate-to-vigorous PA [27] Home equipment and neighborhood aesthetics were strongly associated with leisure-time PA for rural survivors, alongside bicycling infrastructure and proximity to recreational facilities [28] Walking was the preferred PA, with location central to choices, especially in trails, parks, and neighborhoods. [26, 29]

*BE* built Environment, *CP* cancer patient, *SES* Socio Economic Status, *nSES* neighborhood socio economic status, *QoL* quality of life, *PA* physical activity, *AI* artificial intelligence, *nArch* neighborhood archetype

Here Table 4 with more detailed information about each article. Table 2 is added in a different document.

**Table 4:** Summary of Evidence: Associations between Built Environment (BE) variables and cancer outcomes. Summary of Evidence: Associations between Built Environment (BE) variables and cancer outcomes

ID	Author	Country	Population scope	Area of analysis	Population size	Cancer type	Data source	Study design	Main outcome
1	Cheng et al. (2015)	USA	Residence State (California)	Residential area	8995	Breast	3 Case control studies + 2 Cohort prospectives	Cross-sectional (BMI) & Cohort Retrospective (Mortality)	Obesity & Mortality
2	Chu et al. (2022)	USA	Residence City (San Francisco Bay area)	Residential area	774	Bladder, Colon and Leukemia	Patient Registries (APECC; ECHOS-NHL; FOCUS)	Cross-sectional	Health related quality of life (Physical and Mental composite scores)
3	Conroy et al. (2023)	USA	Residence State (California)	Residential area	3975	Breast	Cohort-Prospective	Cohort-Prospective	Cardiovascular Risk
4	De Rouen et al. (2022)	USA	Residence State (California)	Residential area	185,613	Prostate	Patient Registry (California Cancer Registry)	Cohort-Retrospective	Disparities in survival
5	De Rouen et al. (2018)	USA	Residence City (SF Bay area and Los Angeles)	Residential area and Infrastructure	1334	Prostate	Patient Registry (California Cancer Registry)	Cohort-Retrospective	Disparities in survival
6	DeGuzman et al. (2019)	USA	Residence City (Central Virginia)	Residential area	7	Breast and Kidney	2 case–control studies Qualitative	Case Study	Walking as exercise
7	Dioun et al. (2023)	USA	Residence City (New York City)	Metropolitan area, Residential area and Infrastructure	2350	Not focused	Cross-Sectional	Cross-sectional	SARS-CoV-2 infection
8	English et al. (2008)	Canada	Residence City (Greater Toronto Area)	Residential area	14	Breast	Qualitative	Case Study	Types of healing (Physical and mental)
9	Etminani-Ghasrodashti et al. (2022)	USA	National (USA)	Residential area	750	Not focused	Patient Registry (California Cancer Registry)	Cohort-Retrospective	Tumor-free years
10	Etminani-Ghasrodashti et al. (2021)	USA	National (USA)	Residential area	589	Not focused	Cross-Sectional	Cross-sectional	Quality of life (QOL)
11	Hiensch et al. (2020)	The Netherlands	National (The Netherlands)	Residential area	127	Breast	Randomized control trial + 4 years follow up	Cohort-Prospective	Physical activity
12	Jellema et al. (2019)	Belgium, The Netherlands, France and Germany	International (Germany, The Netherlands, France and Belgium)	Infrastructure	7	Not focused	Qualitative	Case Study	In-Cancer-facility patient wellbeing
13	Jones et al. (2015)	USA	National (USA)	Residential area	473	Breast	Cross-Sectional	Cross-sectional	Physical activity
14	Keegan et al. (2014)	USA	Residence State (8 counties in California)	Residential area	4345	Breast	1 Case–control study + 1 Family study	Cross-sectional and Cohort-Retrospective	Recreational physical activity and Survival
15	Lesser et al. (2021)	Canada	National (Canada)	Residential area	114	Not focused	Cross-Sectional	Cross-sectional	Outdoor physical activity
16	Lynch et al. (2010)	Australia	Residence State (Queensland)	Residential area	538	Colon	Cohort-Prospective	Cohort-Prospective	Physical activity
17	McGowan et al. (2017)	Canada	Residence city (Edmonton Alberta)	Residential area	165	Prostate	Randomized control trial	Randomized control trial	Physical activity
18	Papadopoulos et al. (2022)	Canada	Muti-state (Toronto, Ontario and Calgary, Alberta)	Residential area	37	Prostate	Randomized control trial	Cross-sectional and Cohort-Prospective	Physical activity
19	Plascak et al. (2022)	USA	Residence State (New Jersey)	Residential area	57,173	Breast	Patient Registry (New Jersey State Cancer Registry)	Cohort-Retrospective	Disparities in survival/mortality
20	Plascak et al. (2021)	USA	Residence State (New Jersey)	Residential area	476	Breast	Cohort-Prospective	Cohort-Prospective	Perceived stress
21	Robinson et al. (2021)	USA	Residence City (Detroit Michigan)	Residential area	2089	Breast, Prostate and Colon	Cohort-Prospective	Cross-sectional	Body mass index (BMI)
22	Schootman et al. (2020)	USA	Residence City (St. Louis Missouri)	Residential area	215	Breast	Randomized control trial	Cohort-Prospective	Quality of life (QoL)
23	Shariff-Marco et al. (2015)	USA	Residence City (Greater and San Francisco bay areas)	Residential area	5237	Breast	1 Case–control study + 1 Family study	Cohort-Retrospective	Survival/Mortality
24	Shariff-Marco et al. (2017)	USA	Residence State (California)	Residential area	4505	Breast	Cohort-Prospective	Cross-sectional	Body size/ BMI classification
25	Shariff-Marco et al. (2021)	USA	Residence State (California)	Residential area	176,097	Breast	Cohort-Retrospective	Cohort-Retrospective	Survival/Mortality
26	Sposto et al. (2016)	USA	Residence State (California)	Residential area and Infrastructure	12,098	Breast	3 case control studies + 3 cohort prospective	Cohort-Retrospective	Disparities in Mortality
27	Stevens et al. (2023)	USA	Residence State (28 Rural counties in Pennsylvania)	Residential area	219	Not focused	Cross-Sectional	Cross-sectional	Leisure-time physical activity (Intention)
28	Tabatabaie et al. (2020)	USA	Residence City (Denver Metropolitan area)	Residential area	19	Skin	Qualitative	Case Study	Physical activity behavior (intention)
29	Tang et al. (2022)	China	Residence Neighborhood (Yuhui district-Bengbu)	Residential area	Not applicable	Lung	Ecological	Ecological	Survival/Mortality
30	Trinh et al. (2016)	Canada	Residence state (Alberta)	Residential area	432	Kidney	Cross-Sectional	Cross-sectional	Meeting physical activity (PA) guidelines
31	Wu et al. (2018)	USA	Residence City (Los Angeles California)	Residential area	306	Breast	Randomized control trial	Cross-sectional	Self-rated health; Number of co-morbidities; Depressive symptoms

ID	Author	Urbanicity/area of analysis	BE concept/definition	Main BE exposures	Measurement of BE exposures	Outcome definition	Measurement of outcome	Results
1	Cheng et al. (2015)	Urban and Metropolitan suburban; Neighborhood (Ratio 1600 m for REI, 500 m for traffic)	Neighborhood environment	Walkability, Greenness, and Facilities location	Geo-coded address; SES; Pop density; Urbanicity; %Foreign Born; Commuting; Household crowding; Multi-family units; Street-connectivity; Business count; Restaurant Environment Index (REI); Parks	Obesity and Mortality	BMI pre-diagnostic	Attributes of neighborhood environment associated with obesity/mortality, differing across racial groups. For Latinas obesity associated with crowding. For Whites lower nSES associated with obesity/mortality
2	Chu et al. (2022)	Metropolitan, Suburb, City, Town and Rural; Neighborhood (Ratio 1600 m and 500 m)	Neighborhood features attributes	Greenness; Walkability and Facilities location	Racial/ethnic composition; nSES; Population density; housing; Urbanicity; Business data; Farmers markets; Street connectivity; Parks and Traffic density	Health related quality of life (PCS and MCS)	Short-Form healthy survey (SF-12) for Follow-Up care use among survivors (FOCUS) Short-Form healthy survey SF- 36 for Assessment of Patient’s experience of cancer care (APECC) and Experiences of Care and Health Outcomes of survivor (ECHOS)	Higher nSES associated with better PCS. Unhealthy restaurants (REI) associated with worse MCS. Neighborhood archetype variations observed
3	Conroy et al. (2023)	Metropolitan urban, Suburban, City and Small town/rural; Neighborhood (Ratio 1600 m)	Greenness; Neighborhood attributes	Walkability and Facilities location	Geo-coded address: Racial/ethnic composition; nSES; Population density; Immigration; Urbanization; Businesses (REI and RFEI); Commuting by car; Farmers markets; Street connectivity; Parks; Crime index and Traffic density	Cardiovascular Risk	SF-36; Inpatient/ambulatory records of CVD event (MI, heart failure, stroke) with ICD codes	Neighborhood racial/ethnic composition (percent Asian American/Pacific Islander) and crime index were associated with risk of CVD event
4	De Rouen et al. (2022)	Defined in archetypes	Neighborhood Archetypes	Greenness, Walkability and Facilities location	39 Measures characterizing domains of neighborhood social/built environments (Demographics; Immigration; nSES; walkability; residential mobility; Commuting; Rural/urban status; Land use; Food environment)	Disparities in survival	9 Archetypes cluster: New urban/ pedestrian neighborhoods; Upper middle-class neighborhoods; high status neighborhood; City pioneer neighborhood; Suburban pioner neighborhood; Hispanic small town neighborhoods; and Mixed-SES class suburb neighborhoods	Disparities in overall/prostate cancer-specific risk of death by archetype. Highest risk in lower nSES/rural/urban status clusters. Associations varied by race/ethnicity
5	De Rouen et al. (2018)	Not precised	Built Environment factors	Walkability and Facilities location	%Residents traveling by car; Number of businesses; Parks; Farmers Market; nSES; RFEI; REI; Traffic density; Neighborhood housing; Commuting; Residential mobility; Crowding	Disparities in survival	Follow up structured questionnaire: Sociodemographic background; Medical history; Lifestyle factors	African American men had worse survival attenuated by nSES. Increased risk of death associated with lower SES neighborhoods
6	DeGuzman et al. (2019)	Rural, suburban, and small urban	Neighborhood	Greenness and Walkability	Photo-voice with semi-structured questions in interviews about visual cues building and walking paths accessibility and physical safety	Walking as exercise	Qualitative interviews	Visual cues during walks provide recovery motivation. Consistent activity supported by access to buildings/paths. Safety concerns compounded by physical limitations
7	Dioun et al. (2023)	Not precised / Area of residence	Neighborhood level variables and Building characteristics	Walkability	Patient's building characteristics: Assessed value; Residential units; Neighborhood variables (unemployment, racial composition, income, poverty, crowding, density)	SARS-CoV-2 infection	SARS-CoV-2 status from test results or medical record	% Hispanic/Latino population, unemployment, poverty, crowding, and density were associated with SARS-CoV-2 infection
8	English et al. (2008)	Not precised / Healing environments	Therapeutic landscapes	Therapeutic landscapes	Semi-structured interviews with focus on the identification of environments that help with healing	Types of healing (Physical and emotional)	Qualitative interviews	Extraordinary therapeutic landscapes in bodies/homes/community/nature are important. Everyday interactions with these landscapes are most important for healing
9	Etminani-Ghasrodashti et al. (2022)	Not precised / Residence area (Ratio 1 mile)	Built Environment characteristics	Walkability	Employment density; 5D Concept (Density land use street design distance to transit); Survey data: Gas station distance, Treatment center address, Trip frequency	Tumor-free years	Length of tumor-free time period after radiotherapy/chemo treatments	Long travel distance to radiotherapy providers positively associated with greater tumor-free years. Chemo travel distance did not significantly affect tumor-free years
10	Etminani-Ghasrodashti et al. (2021)	Not precised / Residence area	Built Environment characteristics	Walkability	Population density, Entropy index, Transit stop density, Distance to transit, perceived BE and accessibility (questionnaire/Likert scales)	Quality of life (QOL)	Self-reported question evaluating overall QoL after treatment (Likert scale)	BE characteristics contribute to predicting QoL. Travel distance to hospital and perceived accessibility are important predictors
11	Hiensch et al. (2020)	Not precised	Environmental characteristics	Greenness and accessibility	Land use; Sports accommodations; Green and open spaces; Residential area and Number of private recreation facilities	Physical activity	Short questionnaire to assess health-enhancing PA (SQUASH)	Higher baseline leisure/sport PA and more recreational facilities within 1 km buffer correlated with sport/leisure PA levels 4 years post-baseline
12	Jellema et al. (2019)	Not precised / Cancer Center Infrastructure	Built Environment (Cancer Infrastructure)	Architectural design and Accessibility	Autobiographies about experience of cancer treatment (Facility physical features, Furniture, Accessibility)	In-Cancer-facility patient well-being	Autobiographies	Architecture impacts experience of cancer patients. Buildings offer metaphors helping patients rethink experiences of illness and care
13	Jones et al. (2015)	Neighborhood (Scale not specified)	Neighborhood characteristics and Accessibility	Walkability	Self-reported response about barriers (facilities, safety); Neighborhood characteristics; % renters vs homeowners	Physical activity	Self-reported response about lack of interest/facilities; PA 3-item instrument	Relative number of renters vs homeowners associated with lower PA. Individual barriers (interest/space) also associated with lower PA
14	Keegan et al. (2014)	Not precised / Neighborhood (Ratio 1600 m)	Not defined independent variables	Greenness; Walkability and Accessibility	Geo-coded address; Recreation amenities; Farmers markets; Parks; REI; RFEI; Density; Traffic density; nSES; %Total housing units	Recreational physical activity and Survival	Estimation of recreational PA 3 years before diagnosis; Patient medical records for mortality	Women in neighborhoods with no fast food, high traffic, high foreign-born residents less likely to meet PA. Poorer survival associated with lower nSES
15	Lesser et al. (2021)	Not precised / Not related to specific area	Not defined independent variables	Physical environment	Self-reported questionnaire about PA environment barriers facilitators and nature-relatedness (NRS)	Outdoor physical activity	Self-reported questionnaire about PA engagement and psycho-social health	Outdoor environment central for PA choice. Minutes of outdoor PA correlated with subjective happiness and nature relatedness
16	Lynch et al. (2010)	Not precised / related to specific area	Physical environment	Walkability	Interview containing questions about physical environment: lack of facilities perception of safety and unattractiveness	Physical activity	PA measure subscales	Physical environment presented the least salient perceived barriers but was most closely associated with achieving sufficient PA levels 5 months post-diagnosis
17	McGowan et al. (2017)	Not precised / 500 and 1000 m road network buffer	Buffer Walkability	Walkability and Greenness	Postal code buffer walkability (intersection density residential density land use mix) and count of sport complexes	Physical activity	Self-reported minutes/week of PA (Leisure Score Index)	Built environment not associated with self-reported PA and not a contextual effect modifier in PA behavior change intervention
18	Papadopoulos et al. (2022)	Urban / Neighborhood (Not precised)	Neighborhood Environment Walkability Scale	Architectural Design and Accessibility	Neighborhood Environment Walkability Scale (NEWS): Residential density, Proximity to non-residential, Street connectivity, Aesthetics, Safety	Physical activity	Self-report (Godin Leisure-Time) and accelerometer (Actigraph GT3X). PT intervention	Neighborhood aesthetics (sidewalks, trees, lighting) was the strongest predictor of objective MVPA
19	Plascak et al. (2022)	Urban / Not precised	Neighborhood (Disinvestment)	Walkability	Geo-coded address; Observed physical disorder: Garbage/litter Graffiti Burned buildings Dumpsters	Disparities in survival/mortality	Residential physical disorder potential co-founders and tumor prognostic factors	Increases in physical disorder associated with shorter survival time only among women with early stage Breast cancer at diagnosis
20	Plascak et al. (2021)	Not precised / Neighborhood environment	Neighborhood Environment (Physical Disorder)	Walkability	Geo-coded address; 9 characteristics of BE physical disorder and engagement (garbage, graffiti, buildings, yard conditions, sports equipment)	Perceived stress	Cohen's perceived stress scale (PSS-10)	Greater visual cues of engagement marginally associated with lower perceived stress but attenuated in adjusted models. Physical disorder not associated with stress
21	Robinson et al. (2021)	Not precised / Neighborhood (Buffer 1 km)	Neighborhood Walkability Index	Walkability	Multidimensional Neighborhood Walkability Index (NWI): population density, bus-stop density, street connectivity, destination accessibility	Body mass index (BMI)	Self-reported weight/height to calculate BMI	BMI inversely associated with increasing NWI quartile. Inverse relationship observed in men and survivors reporting regular PA
22	Schootman et al. (2020)	Not precised / Residence area	Built Environment	Walkability	Geo-coded address assessing land-use, sidewalks, shoulders, bike lanes, street characteristics	Quality of life (QOL)	8 subscales from RAND 36-Item Short Form Health Survey	BE factors like abandoned buildings/graffiti associated with poorer QoL (emotional role limitations). Sidewalk quality associated with trajectories of QoL subscales
23	Shariff-Marco et al. (2015)	Urban and Rural Residence area (Ratio 1600 m and 500 m)	Neighborhood characteristics	Walkability, Accessibility	Geo-coded address; nSES; pop density; racial composition; traffic; commuting; Business number; Farmers market; RFEI; REI; Recreational facilities	Survival/Mortality	Survival time in days from diagnosis to death/censor	Associations between specific neighborhood characteristics and overall mortality in base models but attenuated in fully adjusted models
24	Shariff-Marco et al. (2017)	Urban and Rural / Neighborhood (1600 m radius)	Neighborhood characteristics	Walkability	Geo-coded address, nSES, pop density, racial composition, traffic, commuting, Business number, RFEI, REI, Parks, street connectivity	Body size/BMI classification	Self-reported height and weight at baseline	Low nSES, high minority composition, high traffic, commuting by car, and fast food restaurants associated with higher odds of overweight/obesity
25	Shariff-Marco et al. (2021)	Urban and Rural / Neighborhood	Neighborhood Archetypes	Accessibility, Walkability	9 archetypal patterns characterizing social/built environments (Demographics, Immigration, nSES, walkability, Land use, Food environment)	Survival/Mortality	Survival time in months	Lowest risk of death in upper middle class suburb; highest in inner city residents. Survival by archetype varied by race/ethnicity
26	Sposto et al. (2016)	Urban and Rural / Not specified	Neighborhood Contextual Factors	Walkability and Greenness	Contextual factors: business count, housing crowding, urban/rural, pop density, public transport, restaurant environment, traffic, hospital SES	Disparities in Mortality	Survival time; PA measures; BMI; Co-morbidity	Contextual, PA, and body size variables influence breast cancer-specific mortality but do not explain racial/ethnic mortality disparity
27	Stevens et al. (2023)	Rural / Residence area	Home Environment and Perceived Built Environment	Walkability and Accessibility	Home environment and Perceived Neighborhood Environment (PA Neighborhood Environment Survey—PANES): Land use, Transit, Infrastructure, Safety, Aesthetics	Leisure-time physical activity (Intention)	Self-reported PA intention	Environmental factors positively associated with LTPA included home environment, perceived support, bicycling infrastructure, recreation facilities, and aesthetics
28	Tabatabaie et al. (2020)	Rural, Small Town and Urban / Outdoor settings	Outdoor settings	Greenness; Walkability	Visual landscape assessment interview (photos of landscapes with trees, shade, maintenance, sidewalk quality)	Physical activity behavior (intention)	Self-reported semi-structured interview	Respondents reported seeking shade/trees to avoid sun exposure. Residential density positively associated with lung cancer mortality (Note: Results text seems mixed with ID 29 in source PDF)
29	Tang et al. (2022)	Urban / District Area	Built Environment	Walkability, Accessibility and Greenness	Land use (density); Road traffic; Spatial form; Blue-green space. Natural environment: Air pollution and Temperature	Survival/Mortality	Lung cancer mortality rates	Lung cancer mortality tends to increase with density of catering/tobacco facilities and road intersections. Mortality decreases when green spaces increase
30	Trinh et al. (2016)	Home Neighborhood (Radius 1 km)	Environment and Built Environment	Greenness; Walkability	Perceived Environment (NEWS); Geo-coded addresses (1 km buffer); Park density; Shopping centers; Recreational centers; Road/Intersection density	Meeting physical activity (PA) guidelines	Modified Godin Leisure-Time Exercise Questionnaire (GLTEQ)	Meeting PA guidelines associated with presence of many retail shops in the neighborhood
31	Wu et al. (2018)	Urban / Neighborhood (Not precised)	Neighborhood Context	Architectural Design, Walkability and Accessibility	Subjective perception of neighborhood stress (Life Stress Scale): Housing, Transportation, Public services, Crime	Self-rated health; Co-morbidities; Depressive symptoms	Short-Form-36; CES-D scale	Greater neighborhood stress associated with poorer self-reported health, more co-morbidities, and depressive symptoms

### BE variables categorization

The initial intention of this systematic review was to identify BE components operating at both the urban and architectural levels that may influence CP during the extended treatment period, a phase when the OI is most directly experienced. However, throughout the synthesis process, a recurrent theme emerged: density and household crowding appeared across numerous studies as distinct determinants of patients’ experiences and outcomes. This finding revealed the need to introduce a third analytical dimension beyond the traditional urban–architectural dichotomy. Accordingly, the reviewed variables were intentionally grouped into three main categories:

- Density-related (DRV): density and house crowding.
- Architecture-related (ARV): aesthetics, design, and indoor environment.
- Urban-related (URV): transport/traffic, walkability, street connectivity, food environment, retail environment, recreational environment, facilities environment, businesses environment, green areas/nature, and pedestrian infrastructure

### Risk of Bias assessment

The Newcastle–Ottawa tool assessment revealed a generally low risk of bias, with 21 of 29 analyses (from 19 of 26 articles) receiving the maximum score (6–9 points). Key sources of bias were sample representativeness (Q1) and lack of objective outcome measures (Q6), particularly in studies measuring BMI, PA, and QoL. The full results of the risk of bias assessment are reported in Supplementary Table S1.

## Discussion

This review provides an overview of the evidence linking the BE with health outcomes in CP, highlighting a limited yet complex body of research. The scarcity of studies is further complex as for the significant variability among authors in defining BE variables, as some include both social and physical environments, while others focus solely on one aspect. This adaptive nature of the BE concept and its variables complicate comparisons and synthesis of results.

In this review, the primary goal was to identify BE variables that are particularly relevant during the extended treatment period. Accordingly, the findings were intentionally grouped into three main categories (DRV, URV, ARC) developed to analyze features that specifically impact CP who spend significant and repeated periods of time in or around the OI and their immediate residential environments. By structuring the review in this way, it bridges the gap between general BE features commonly studied in population-based or preventive research and those most pertinent to patient experience and outcomes during treatment.

The 31 reviewed articles highlight how certain BE characteristics influence QoL, well-being, mental health, and PA in among CP and survivors. Notably, over half were published in the past four years, reflecting a growing interdisciplinary approach to the patient in epidemiological research.

Previous systematic reviews have highlighted the importance of the geographic location of the patients’ residence and identified that the urban/rural location of the house [44–46] and increased travel time/distance [47] as significant BE variables that worse cancer survival for rural/remote CP [44] and impact negatively the stage at diagnosis, treatment adherence, prognosis, and QoL [47].

In our review, DRV consistently showed direct associations with comorbidities [18], higher waist-to-hip ratio [20], lower QoL [14], and lower levels of PA [48]. These findings reflect urban trends showing that high-density environments are often more harmful [49, 50]. However, for CP, understanding the BE is particularly important, as evidence from general populations suggests that high density when integrated with thoughtful urban and architectural planning (e.g., mixed land use, access to transit, green spaces, and social equity) can mitigate negative effects and promote health and well-being [51].

When discussing density and cancer outcomes, many authors focus on physical space limitations. Some hypothesize that higher mortality in dense areas results from limited open space, reducing opportunities for PA and increasing cancer risks [38]. Others argue that high traffic density, combined with poor pedestrian safety, exacerbates this issue by discouraging PA and limiting treatment adherence [19]. These perspectives highlight how density not only constrains space but also interacts with the broader urban structure, shaping how patients move through and experience their surroundings.

Building on this relationship between spatial constraint and urban form, the next group of variables (URV) was created to capture the functional and design characteristics of the urban environment that mediate daily patient behaviors and exposures. Several studies in this review analyzed open spaces, such as sidewalks and parks. Although analyzed separately across different studies, the findings consistently emphasize their interconnectedness. Identified characteristics of green spaces, such as trees, shade, sidewalks, and maintenance, may act as behavior modifiers for cancer survivors, influencing their PA decisions, sun-exposure behaviors, and QoL trajectory [30, 32]. Furthermore, since such spaces have been shown to mitigate anxiety and depression [52], identifying URV is essential for understanding their therapeutic potential for CP and for informing strategies to transform the urban landscape toward health-promoting environments.

Neighborhoods with mixed land use and diverse services have been associated with improved walkability and PA among CP and survivors [53, 54]. Yet, without proper regulation, such areas may develop unhealthy retail clusters, increasing BMI and related risks [19, 39, 55], further reinforcing the URV–health connection and illustrating the complexity of urban influences on patient outcomes.

Other URV, particularly those embedded in vehicle-oriented infrastructures, can create additional barriers for CP. Such settings expose individuals to environmental stressors like pollution, noise, and unsafe transit conditions. The reviewed literature identified that current situation on traffic density [20, 21, 24, 25, 39], distance to transit [14, 37], transit accessibility [28, 56], and commuting [21, 25, 57, 58] affect negatively patient mobility and access to care.

Similarly, lower access to private vehicles and inadequate public transit not only increase disparities in cancer care [59] but also shape the lived experience of treatment. For many patients, the simple act of traveling to care becomes a physical and psychological burden especially during prolonged treatment regimens that require repeated visits. These barriers influence treatment adherence and decision-making, often leading patients to select less intensive or less frequent regimens [60] which may compromise outcomes. The stress associated with long or unreliable commutes can be a contributor to treatment discontinuation, emotional exhaustion, decreased perceived quality of care, delayed diagnosis, and poorer survival outcomes [61].

While the current trend toward centralizing oncology services seeks to improve clinical quality, it can unintentionally exacerbate patient distress when accessibility is not addressed through supportive design and transport infrastructure [62]. From a patient-centered perspective, these findings underscore that URV shapes experiential dimensions of care, directly affecting comfort, autonomy, and continuity during treatment.

Transitioning from the urban scale to the architectural scale, the reviewed articles paid comparatively less attention to ARV despite several authors recognizing their potential impact on health outcomes and patient well-being, particularly within healthcare facilities [63]. Clinical spaces designed primarily for procedural efficiency may unintentionally restrict mobility and increase stress during treatment, affecting both physical and psycho-spiritual well-being during treatment [33].

At the design scale, aesthetic and sensory attributes (cleanliness, order, natural light, views) also contribute to emotional well-being and lower stress among cancer patients [22, 59]. Comparable findings in general populations link physical disorder (graffiti, litter, poor aesthetics) with higher stress, substance use, and reduced PA [64–67]. Further exploration could reveal how these architectural and design variables uniquely shape the daily experiences and recovery trajectories of cancer patients.

To our knowledge, this is the first systematic synthesis focusing on BE variables, particularly urban and architectural, affecting CP during the treatment phase. Despite limited research, these findings clarify how DRV, URV, and ARC features intersect to shape patients’ daily experiences. However, heterogeneity in study design, BE metrics, and definitions continues to limit comparability. Conceptual inconsistencies, exclusion of neighborhood socioeconomic status (nSES) as a BE variable, and the predominance of studies from high-income countries further constrain generalizability. Still, these results provide a critical foundation for developing future BE frameworks tailored to cancer care settings and diverse contexts.

## Conclusions

This systematic review highlights the limited but growing evidence on how specific features of the BE influence CP outcomes during the extended treatment period, an often-overlooked phase in which the OI is most directly experienced. The findings reveal that while research remains fragmented and conceptually heterogeneous, the BE plays a measurable role in shaping QoL, mental health, and PA among CP and survivors.

A central contribution of this review was the categorization of BE variables into density-related (DRV), urban-related (URV), and architectural-related (ARV) dimensions, enabling a more patient-centered reading of the physical environment. DRV capture the human spatial constraints and social exposure of dense living conditions; URV describe mobility, accessibility, and environmental quality; and ARV encompass the sensory and design attributes of healthcare and residential spaces. Together, these dimensions provide a framework that situates the patient within the multi-scalar physical contexts that modulate both treatment journey experience and health outcomes.

Although BE features such as open spaces, diverse land use, and aesthetically favorable environments appear to improve QoL and PA, the lack of conceptual and methodological standardization continues to hinder cross-study comparison and meta-analytical synthesis. Establishing unified definitions and standardized variables is therefore essential to integrating BE research into cancer epidemiology. Such integration would extend beyond the traditional biomedical paradigm incorporating a biopsychosocial and spatial perspective that recognizes the BE not merely as a background, but as an active determinant of treatment experience in cancer care.

This approach reframes the BE as a therapeutic environment, where architectural and urban dimensions converge to support patient treatment. It also underscores the need for interdisciplinary collaboration between oncology, environmental psychology, public health, and spatial sciences to capture how built environments affect physiological stress, cognitive load, and recovery trajectories during prolonged treatment engagement. 

## Future directions

Future research on the CP journey should prioritize the standardization of BE variables and the development of spatially explicit frameworks capable of quantifying how physical environments interact with patient behavior and well-being throughout the cancer treatment continuum. A critical shift is required in how the spatial disciplines engages with Evidence -Based Design (EBD). While EBD has established a foundation for using research to inform design, the current evidence results in an open loop: evidence is “consumed” but the environments are rarely measured post implementation. Future studies must prioritize post-ocuppancy evaluation as a standard clinical and architectural protocol. To achieve this, Longitudinal and mixed-method study designs, integrating geographic information systems (GIS), wearable sensors, and recently developed software for spatial mapping to optimize the BE of cancer care.

Emerging technologies offer transformative opportunities. Artificial Intelligence (AI)  machine learning (ML) can be applied to model spatial complexity, detect BE–health correlations across large datasets, and identify predictive environmental signatures associated with CP well-being. Likewise, spatial analytics and digital twins allow researchers to simulate and optimize oncology environments before implementation or modification. Moreover, cross-cultural and survivorship-focused comparisons will be crucial to understanding how cultural, social, and economic contexts influence the CP experience in the BE.

In sum, advancing BE research in oncology requires a neuro-spatial and technological turn: one that integrates architecture, urban design, behavioral science, and data-driven modeling to create evidence-based, patient-centered environments. Such an approach would not only inform public health policy and facility planning but also redefine the BE as a measurable, modifiable ally of cancer treatment and survivorship.

## Competing interests

The authors declare no competing interests.

## Supplementary Information

Below is the link to the electronic supplementary material.

Supplementary file1 (PDF 38 kb)

Supplementary file3 (PDF 45 kb)

## Data Availability

No datasets were generated or analysed during the current study.

## Abbreviations

ARV: Architecture-Related Variables
BE: Built environment
CDIs: Cancer-Dedicated Infrastructures
CP: Cancer patient
DRV: Density-Related Variables
nSES: Neighborhood socio economic status
OI: Oncology Infrastructure
PA: Physical Activity
QoL: Quality of Life
SES: Socioeconomic Status
URV: Urban-Related Variables
AI: Artificial intelligence
