# Supplementary material for: Built environment (BE) and cancer: a systematic review of the BE’s impact during the treatment journey and patient outcomes
Source: Cancer Causes Control. 2026 May 26;37(6):89. doi: 10.1007/s10552-026-02132-5 (PMC13212709; doi:10.1007/s10552-026-02132-5)
Supplement: Supplementary file 1 — Supplementary file1 (PDF 38 kb) [file 10552_2026_2132_MOESM1_ESM.pdf]

"Newcastle-Ottawa Scale" (NOS) APPLICATION

Note: A study can be awarded a maximum of one star for each numbered item within the Selection and Outcome categories. A maximum of two stars can be given for Comparability

**Selection**	**1pt**		**0pt**	
Q1) Representativeness of the exposed cohort	a) truly representative of the average in the community	b) somewhat representative of the average in the community	c) selected group of users eg nurses, volunteers	d) no description of the derivation of the cohort
Q2) Selection of the non exposed cohort	a) drawn from the same community as the exposed cohort		b) drawn from a different source	c) no description of the derivation of the non exposed cohort
Q3) Ascertainment of exposure	a) secure record	b) structured interview	c) written self report	d) no description
Q4) Demonstration that outcome of interest was not present at start of study	a) yes		b) no	
**Comparability**	**1pt**		**0pt**	
Q1) Comparability of cohorts on the basis of the design or analysis	a) study controls for		b) study controls for any additional factor	
**Outcome**	**1pt**		**0pt**	
Q1) Assessment of outcome	a) independent blind assessment	b) record linkage Ø	c) self report	d) no description
Q2) Was follow-up long enough for outcomes to occur	a) yes		b) no	
Q3) Adequacy of follow up of cohorts	a) complete follow up - all subjects accounted for	b) subjects lost to follow up unlikely to introduce bias - small number lost - >80 % follow up, or description provided of those lost	c) follow up rate < 80%and no description of those lost	d) no statement

Appendix 1 ' NOS APPLICATION FILTERING.
