# Supplementary material for: Built environment (BE) and cancer: a systematic review of the BE’s impact during the treatment journey and patient outcomes
Source: Cancer Causes Control. 2026 May 26;37(6):89. doi: 10.1007/s10552-026-02132-5 (PMC13212709; doi:10.1007/s10552-026-02132-5)
Supplement: Supplementary file 2 — Supplementary file3 (PDF 45 kb) [file 10552_2026_2132_MOESM2_ESM.pdf]

DETAILED RESULTS OF THE "Newcastle-Ottawa Scale" (NOS) APPLICATION

Author	Study design	Selection				Comparability	Outcome			Sub-total Selection	Sub-total Comparability	Sub-total Outcome	TOTAL
		Q1	Q2	Q3	Q4	Q1	Q1	Q2	Q3				
**Cheng et al. T2**	**Cohort Retrospective (M)**	1	1	1	1	2	1	1	1	4	2	3	**9**
Conroy et al.	Cohort Prospective	1	1	1	1	2	1	1	1	4	2	3	**9**
De Rouen et al.	Cohort Retrospective	1	1	1	1	2	1	1	1	4	2	3	**9**
De Rouen et al.	Cohort Retrospective	1	1	1	1	2	1	1	1	4	2	3	**9**
Etminani-Ghasrodashti	Cohort Retrospective	1	1	1	1	2	1	1	1	4	2	3	**9**
Keegan et al. T1	Cohort Retrospective	1	1	1	1	2	1	1	1	4	2	3	**9**
Lynch et al.	Cohort Prospective	1	1	1	1	2	1	1	1	4	2	3	**9**
Papadopoulos et al. T1	Cohort Prospective	1	1	1	1	2	1	1	1	4	2	3	**9**
Plascak et al.	Cohort Retrospective	1	1	1	1	2	1	1	1	4	2	3	**9**
Shariff-Marco et al.	Cohort Retrospective	1	1	1	1	2	1	1	1	4	2	3	**9**
Shariff-Marco et al.	Cohort Retrospective	1	1	1	1	2	1	1	1	4	2	3	**9**
Sposto et al.	Cohort Retrospective	1	1	1	1	2	1	1	1	4	2	3	**9**
Hiensch et al.	Cohort Prospective	1	1	1	n.a.	2	?	1	1	3	2	2	**7**
McGowan et al.	Randomized control trial	1	1	1	n.a.	2	0	1	1	3	2	2	**7**
Plascak et al.	Cohort Prospective	1	1	1	n.a.	2	0	1	1	3	2	2	**7**
Schootman et al.	Cohort Prospective	1	1	1	n.a.	2	0	1	1	3	2	2	**7**
Chu et al.	Cross-sectional	1	1	1	n.a	2	1	n.a	n.a	3	2	1	**6**
Dioun et al.	Cross-sectional	1	1	1	n.a	2	1	n.a	n.a	3	2	1	**6**
Etminani-Ghasrodashti	Cross-sectional	1	1	1	n.a	2	1	n.a	n.a	3	2	1	**6**
Jones et al.	Cross-sectional	1	1	1	n.a	2	1	n.a	n.a	3	2	1	**6**
Keegan et al. T2	Cross-sectional	1	1	1	n.a	2	1	n.a	n.a	3	2	1	**6**
Papadopoulos et al. T2	Cross-sectional	1	1	1	n.a	2	1	n.a	n.a	3	2	1	**6**
Cheng et al. T1	Cross-sectional (BMI)	1	1	1	n.a.	2	0	n.a	n.a	3	2	0	**5**
Robinson et al.	Cross-sectional	1	1	1	n.a	2	0	n.a	n.a	3	2	0	**5**
Shariff-Marco et al.	Cross-sectional	1	1	1	n.a	2	0	n.a	n.a	3	2	0	**5**
Stevens et al.	Cross-sectional	1	1	1	n.a	2	0	n.a	n.a	3	2	0	**5**
Trinh et al.	Cross-sectional	1	1	1	n.a	1	1	n.a	n.a	3	1	1	**5**
Wu et al.	Cross-sectional	1	1	1	n.a	2	0	n.a	n.a	3	2	0	**5**
Lesser et al.	Cross-sectional	0	1	1	n.a	2	0	n.a	n.a	2	2	0	**4**
DeGuzman et al.	Case Study	NOT APPLICABLE											**0**
English et al.	Case Study	NOT APPLICABLE											**0**
Jellema et al.	Case Study	NOT APPLICABLE											**0**
Tabatabaie et al.	Case Study	NOT APPLICABLE											**0**
Tang et al.	Ecological	NOT APPLICABLE											**0**

Table S1 Legend: Assessment of Risk of Bias in Primary Studies using the Newcastle-Ottawa Scale (NOS). The methodological quality of the observational studies was evaluated using the Newcastle-Ottawa Scale (NOS), which assigns star points (score of 1 or 0) across three domains for a maximum total score of 9 stars (higher score indicates lower risk of bias). The columns in the table correspond to the individual questions (Q) within each quality category of the NOS. The following symbols are used: 1 (Criterion met), 0 (Criterion not met), NA (Not Applicable), and ? (Unclear/Not reported). Note: Articles identified with designations such as T1 or T2 represent distinct studies or separate analyses (e.g., different exposures or cohorts) included within the same published article, which were assessed independently. The scoring criteria and the complete format of the NOS questions for cohort and cross-sectional studies are detailed in Appendix 1 of this document.
